# Development of low-cost, compact chiroptical imaging systems†

**DOI:** 10.1039/d4nr01651c

**Published:** 2024-06-04

**Authors:** Matthew D. Ward, Ronan Docherty, Louis Minion, Xingyuan Shi, Kai Anson, Giuliano Siligardi, Jenny Nelson, Jessica Wade, Matthew J. Fuchter

**Affiliations:** a Department of Physics, Imperial College London South Kensington Campus Prince Consort Road London SW7 2AZ UK; b Centre for Processable Electronics, Imperial College London South Kensington Campus London SW7 2AZ UK; c Department of Materials, Exhibition Road, Imperial College London South Kensington Campus London SW7 2AZ UK jessica.wade@imperial.ac.uk; d Department of Chemistry and Molecular Sciences Research Hub, Imperial College London White City Campus 82 Wood Lane London W12 0BZ UK m.fuchter@imperial.ac.uk; e B23 Beamline, Diamond Light Source Ltd Harwell Science and Innovation Campus Didcot UK

## Abstract

Circular dichroism spectroscopy is a key probe of the structural and optical properties of chiral materials, however, commercial circular dichroism spectrometers are large, prohibitively expensive and rarely offer environmental control of the sample under test. Using Fresnel rhombs as inexpensive broadband quarter-wave plates, we demonstrate two novel, low-cost (<£2000) and portable imaging systems controlled by our own bespoke open-source control software which are capable of spatially mapping the circular dichroism of chiral solid state films. By coupling these imaging systems with a temperature controlled stage, we show that we can rapidly identify the thermal processing conditions required to maximise circular dichroism in chiral solid state films by measuring circular dichroism *in situ* during thermal annealing of a sample under test. The accuracy and spatial resolution of these circular dichroism imagers are cross-compared against our previous studies using an existing circular dichroism imaging system at the Diamond Light Source and are shown to be in good agreement, with a sensitivity down to 250 mdeg and a spatial resolution of 100 μm.

## Abstract 2

Real-time circularly polarised imaging is achieved using two low-cost (<£2000) and compact imaging systems controlled by open-source python software.

## Graphical abstract

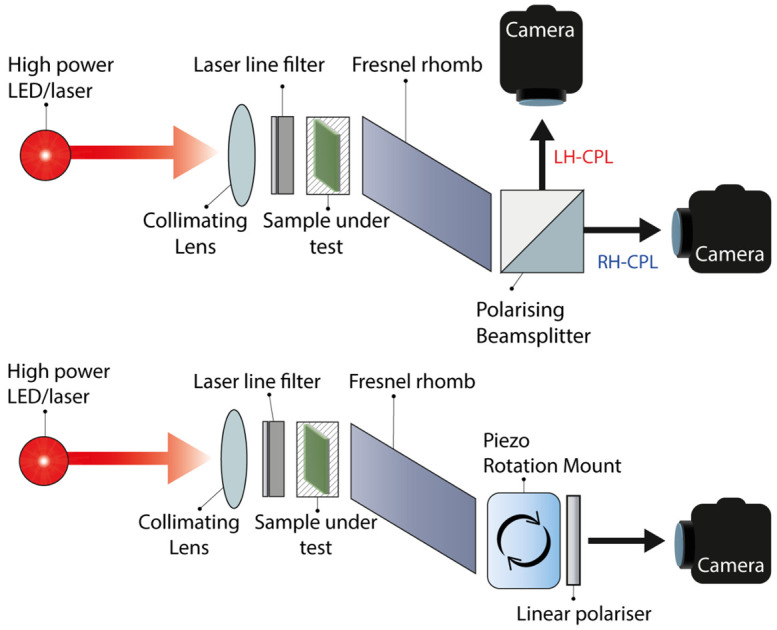

## 1 Introduction

The chiral optical (chiroptical) properties of chiral functional materials will transform future technologies; including data storage,^1^ biosensing,^2^ quantum computation,^3,4^ environmental monitoring,^5^ and next-generation displays.^6^ These exciting prospective applications have driven considerable research interest in the design and study of chiral materials.^7–9^ Measurements of the differential absorption and emission of left- and right-handed circularly polarised light (circular dichroism, CD, and circularly polarised luminescence, respectively) present an essential tool for investigating the chiroptical response. The results of these experiments are critical for the design of innovative materials and devices. The majority of approaches to characterise the chiroptical response are expensive, which precludes them from widespread use. These pieces of equipment are rarely compact or modular, making *in situ* measurements challenging and restricting their location to dedicated characterisation laboratories. For example, commercially available CD spectropolarimeters require considerable bench space, cannot provide spatially resolved information, are difficult to adapt for *operando* characterisation and typically require expensive accessories for custom measurements.^10^ Further, the software required to acquire and analyse spectra is rarely open source, with costly licenses and upkeep fees.

Spatially resolved CD measurements are essential for understanding and optimising the structural organisation of functional materials. They can be used in the development of fabrication protocols and find the conditions that lead to a homogeneous chiroptical response across a thin film, which is key for the realisation of defect-free devices that make use of circularly polarised light (CPL). A summary of CD imaging systems that allow for such spatially resolved measurements reported in the literature is provided in Table 1.

**Table 1:** Comparison of previously published CD-imaging systems, including spatial and CD resolution. “—” indicates a figure of merit which has not been reported by the authors

Description	Spatial res.	CD res. (mdeg)	Comments	Ref.
(a) Photoelastically modulated input light adapted to a scanning confocal microscope	200 nm	—	High spatial resolution and sensitivity. Affected by signals originating from sinusoidal polarisation modulation and sample linear birefringence/dichroism.^11,12^ System used for the *in vitro* study of the change in the structure of chromatin during sperm development in Drosophila	13 and 14
(b) Commercial microscope, wave plate, and linear polariser. Spectrograph and cooled CCD detector enable spectrally resolved CD measurements	500 nm	90–110	Avoids problems associated with electronic modulation but complicated calibration procedure and requires commercial microscope. System used for the study of 1,8-dihydroxyanthraquinone crystals (2.5 mm × 2.5 mm × 100 μm), chiral poly(9,9-bis[(3*S*)-3,7-dimethyloctyl]fluorenyl-2,7-diyl-*alt*-benzo[2,1,3]thiadiazol-4,8-diyl) films (80 × 60 μm^2^) and phenylene bis-thiophenyl propynone films (950 × 700 μm^2^)	15–17
(c) Collimated synchotron radiation microbeam, piezoelectric stage, and photoelastic modulator	50 μm	<1	High sensitivity enabled by synchrotron radiation, but access is restricted to synchrotron users and raster scanning technique is slow. System used for various chiropotical materials, such as cellulose nanocrystal films (2 mm × 2 mm × 90 μm)	18
(d) Laser scanning confocal microscope adapted with a photoelastic modulator	<1 μm	—	High sensitivity afforded by collimated laser, but requires expensive commercial scanning microscope. System used to study films of chiral poly[9,9-bis((3*S*)-3,7-dimethyloctyl)-2,7-fluorene] with minimum feature sizes of ∼1 μm	19
(e) Scanning near-field optical collection mode microscope combined with photoelastic modulator	∼100 nm	—	Beyond diffraction limit resolution, but requires complex and expensive instrumentation. System used to image a two-dimensional chiral pair of nanostructures (∼1.2 μm × 0.7 μm)	20
(f) Nanostructured chiral meta-lens designed to separate left and right circularly polarised light	5 μm	—	High sensitivity, but requires a bespoke meta-material lens and restricted to highly specialist users. System used to image the exoskeleton of a chiral beetle, *Chrysina gloriosa* (∼10 mm × 10 mm)	21
(g) Direct comparison of microscope images captured under L- and R-CPL illumination using a traditional polarisation optical microscope with a quarter waveplate	∼5 μm	<130	High spatial resolution, but slow data acquisition. Requires manual rotation of the quarter waveplate and 3–10 images per polarisation, leading to a low temporal resolution of 1.25 minute per mm^2^. System used to image self-assembled helical aggregates of liquid crystal – based gold nanoparticles with minimum feature sizes of ∼5 μm	22
(h) Commercial microscope optics using two beam displacers and a chopper to modulate the handedness of CPL illuminating the sample under test	300–400 nm	∼20	High spatial resolution. The use of beam displacers and an optical chopper in place of a PEM eliminates CD artefacts arising from linear birefringence and linear dichroism in the samples under test. System used to image a two-dimensional array of chiral (swirl-shaped) gold nanostructures (∼1 μm × 1 μm)	23
(i) Far-field extinction microscope making use of a supercontinuum laser as the light source with a polarising beamsplitter and liquid crystal retarder to modulate the handedness of CPL transmitted to the sample under test	∼500 nm	∼1	High spatial resolution but costly components, such as the supercontinuum laser. System used to image chiral plasmonic nanostructures (150 nm × 150 nm) and chiral crystal mercury sulfide nanoparticles (80 nm diameter)	24
(j) This work	100 μm	250	Compact, low-cost CD imaging with high temporal resolution of 0.2 s. Entry-level system with open-source control software. Upgradable CD sensitivity and spatial resolution of 250 mdeg and 100 μm, respectively	

The first CD microscopes were constructed by Maestre and co-workers in the 1980s, however, the use of imperfect electronic polarisation modulation techniques led to parasitic artefacts affecting their measurements (Table 1, row a).^13,14^ In an attempt to mitigate these issues, Kahr and co-workers proposed a visible-light microscope based on mechanical modulation – but the measurement process still resulted in artefacts appearing in the CD signals (Table 1, row b).^15,25^ Recently, CD imaging using the collimated beamlight of synchrotron facilities has enabled high resolution spatially resolved chiroptical measurements, but accessing synchrotron facilities is non-trivial, measurements are time consuming and synchrotron operating costs are considerable (Table 1, row c).^18,26–28^ Additionally, CD spectra can only be acquired across a limited spectral range (200–650 nm), which renders the characterisation of emerging materials (*e.g.* low-bandgap non-fullerene acceptors) impossible. Characterisation in the infrared is critical for the realisation of highly selective CPL photodetectors for efficient, next-generation optical communications which make use of CPL to double communication bandwidth.^29,30^ It should be noted that of the custom systems reported to date (Table 1), none provide access to the open source software required to operate the instrument.

CD measurements as a function of temperature allow users to monitor different phases within their materials and identify the optimum processing conditions to maximise the chiroptical response.^31^ Additionally, chiroptical imaging systems capable of making non-destructive measurements *in situ*/*operando i.e.*, under conditions relevant for device operation, can help to uncover the fundamental mechanisms responsible for CPL emission and spin-selective charge transport. Measurements at high and low temperatures can more accurately replicate the optical responses of functional materials as the active layers of real-world devices. For example, light-emitting diodes (LEDs) have operating temperatures of ∼85 °C, while spin valves typically require cryogenic cooling. It is therefore inappropriate to characterise only the room temperature CD response when attempting to determine structure–performance relationships. Finally, the rapid acquisition of CD spectra during chemical synthesis can permit the design of materials with desired spectral features. As such, the design of a low-cost, compact, and modular CD spectropolarimeter is of considerable research interest.

Here we present an alternative approach to the chiroptical characterisation of functional materials. The entire apparatus can be assembled at a low cost (<£2000), and operates using open source software based on free Python libraries. We demonstrate that the instrument is capable of measuring CD signals as weak as 250 mdeg and visualising features as small as 100 μm. The system is entirely modular, allowing for the integration of stages, allowing for temperature or environmental control, and accommodating upgrades to light sources or detectors as they become more readily available. All custom components (*e.g.* the housing and sample holders) can be 3D printed and assembled on any surface, removing the need for an optical table and allowing this tool to be used in any lab setting. The low cost and portability of this system also make it suitable for Physics and Chemistry education and outreach activities to visualise chirality on the macro- and micro-scale. Despite the low cost and small size of this system, the measured ellipticity (for values above the lower limit of 250 mdeg) is almost identical to that measured using a benchtop spectropolarimeter.

## 2 Circular dichroism

Circular dichroism is defined as the differential absorbance of left- and right-handed light (L- and R-CPL, respectively). This can be expressed directly as a difference in absorbance:

1 Δ*A* = *A*_L_ − *A*_R_

or, more commonly, in millidegrees of ellipticity (often referred to as simply “CD”):2
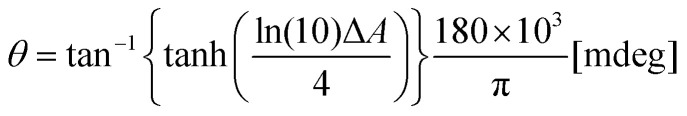
which, for Δ*A* ≪ 1, simplifies to

3 *θ* = 32 982 × Δ*A* [mdeg].

A full derivation of eqn (2) and (3) are provided in the ESI† alongside a description of the range of Δ*A* values over which the approximate form is valid (Fig. S1 and S2†).

Typically, ellipticity is measured by irradiating a sample with monochromatic unpolarised light and evaluating the difference in the transmitted L- and R-CPL intensity (*I*_L_ and *I*_R_, respectively). The *A*_L_ and *A*_R_ for the sample are defined as4
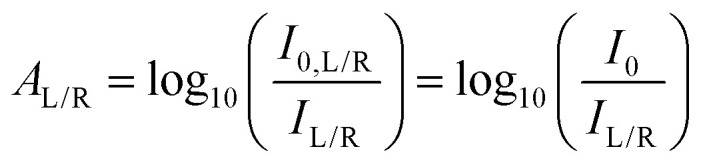
where we have assumed that the incident intensity of L-CPL (*I*_0,L_) and R-CPL (*I*_0,R_) are equal (*I*_0_). Applying eqn (4)–(1), we obtain:5
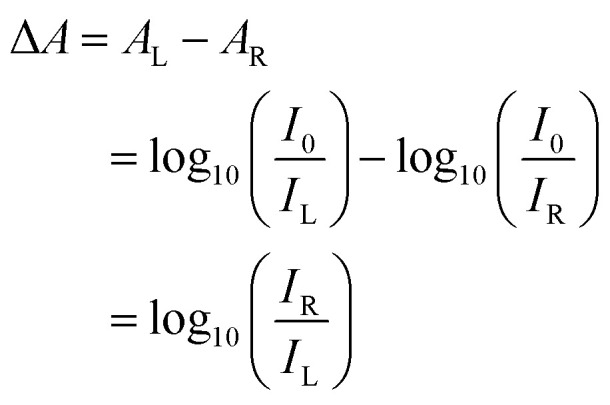


Eqn (5) indicates that Δ*A* (and ellipticity, through eqn (2)) can be determined by measuring only *I*_L_ and *I*_R_, that is, without measurement of the incident intensity, *I*_0_.

To measure *I*_L_ and *I*_R_, L- and R-CPL must be selectively filtered, which is often achieved by the combination of a quarter-wave plate (QWP) and a linear polariser (LP). The QWP converts L- and R-CPL into two distinct orthogonal LPs, and the LP is rotated to select one of these linear polarisations to be transmitted to a detector for the measurement of *I*_L_ or *I*_R_ (ESI, Fig. S3†), enabling the calculation of Δ*A*.

## 3 Imaging system design

**Fig. 1:**
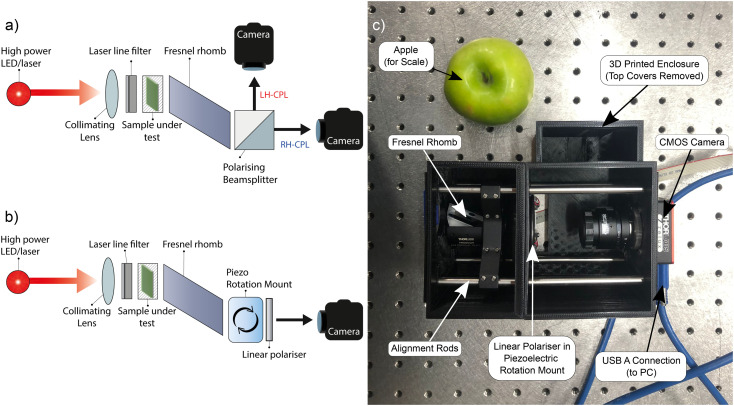
Schematics of the two- (a) and one-camera (b) circular dichroism imaging systems developed in this work alongside a photograph of the optimised system (c) with a black 3D printed enclosure to block stray light and enable system portability.

We developed two simple CD imaging systems (Fig. 1). Collimated monochromatic light from a high-power LED or laser is passed through a sample. A Fresnel rhomb is used to separate the transmitted L- and R-CPL into two linear polarisations. Fresnel rhomb retarders feature broadband performance (400–1550 nm) at a remarkably low cost (<£400). Alternatives such as broadband quartz QWPs and photoelastic modulators were considered; however, both are prohibitively expensive (>£2000 and >£10 000, respectively). Additionally, the 34.8 Hz frame rate of the low-cost camera used in this setup is slower than the 50 kHz switching speed of a photoelastic modulator, rendering them unsuitable for this application.

The two-camera circular dichroism imager (2-CCD*i*), uses a polarising beamsplitter to direct the two linear polarisations from the Fresnel rhomb (corresponding to L-CPL and R-CPL) to two separate monochrome CMOS cameras. Our open-source software allows for L- and R-CPL images of the sample to be viewed independently, digitally aligned to account for small discrepancies in camera alignment and, using eqn (2) and (5), generate images of spatially mapped Δ*A* and ellipticity. The one-camera circular dichroism imager (1-CCD*i*) uses an LP mounted on a piezoelectric rotation mount at the output of the Fresnel rhomb. The piezoelectric rotation mount is capable of rotating the LP at a rate of 430° s^−1^, enabling the LP to rapidly switch between L- and R-CPL in approximately 0.2 s. To map the Δ*A* and ellipticity, the L-CPL and R-CPL images are acquired sequentially. The software displays images of the sample under L- and R-CPL transmission, mapped Δ*A* (eqn (5)), and ellipticity (eqn (2)) in real time.

For the case of the 1-CCD*i*, given that a LP is rotated to acquire images of L- and R-CPL transmission, care should be taken to ensure that the camera does not contain intrinsic aligned elements sensitive to linearly polarised light. Such elements may lead to apparent CD signals in samples without true CD. This can be straightforwardly tested by acquiring a CD map in the absence of a sample – an artefact-free imaging system will yield a CD map of ≈0 mdeg.

The 1-CCD*i* has considerable advantages over the 2-CCD*i* counterpart. The 2-CCD*i* is less compact, requires two cameras on the perpendicular arms of the beamsplitter, and is more complicated to assemble as care must be taken to ensure identical path lengths/sample imaging areas for the two cameras. Even with careful assembly, post-processing is required to align the L- and R-CPL images for reliable calculations of Δ*A*. Additionally, the 2-CCD*i* cannot resolve small asymmetries in the photoresponses of individual pixels within the two cameras, which may lead to artefacts in the measurement of Δ*A*. The 1-CCD*i*, on the other hand, does not require co-alignment, and identical pixel responsivity under L- and R-CPL illumination is guaranteed. Finally, the extinction ratio of the transmitted beam (>1000 : 1) and reflected beam (∼100 : 1) of the beamsplitter in the 2-CCD*i* are different, meaning that the purity of linear polarisation transmitted to each CCD will differ for the transmission and reflection case. One shortcoming of the 1-CCD*i* approach is the delay of 0.2 s between the L- and R-CPL images – the 2-CCD*i* systems can capture L- and R-CPL images simultaneously – but this acquisition time is comparable to most commercial instruments. Although the extinction ratio of the wire grid linear polariser used in the 1-CCD*i* (>800 : 1) is lower than a number of alternatives, such as the Glan–Taylor polariser (100 000 : 1), this option was selected for its lower cost (£223.78) at the chosen aperture of 25.0 mm, compared to the Glan–Taylor polariser with the largest aperture available (GT15, Thorlabs, £1054.28).

These imaging systems permit the measurement of the sample under ambient conditions, as well as in chambers with environmental control. These can allow for measurements as a function of temperature and in conditions relevant for device operation at a fixed wavelength. Further, replacing the LED or laser in these systems with a tuneable light source could allow for the acquisition of spectrally resolved information.

For both imaging systems, the spatial mapping of *g*_abs_ should be avoided, since cameras measure the transmission rather than the absorption of CPL. This is discussed in detail in the ESI.†

## 4 Results and discussion

To evaluate the performance of our compact CCD*i* systems, we fabricated thin films of poly(9,9-dioctylfluorene-*alt*-benzothiadiazole) (F8BT hereafter) blended with enantiopure [*P*]- and [*M*]-1-aza[6]helicene ([*P*]- and [*M*]-aza[6]H hereafter) which have been extensively studied by our group.^31–35^ When spin-coated into solid state films, F8BT:[*P*]- and F8BT:[*M*]-aza[6]H demonstrate negligible ellipticity (<100 mdeg) in the absorption band of F8BT in their “as-cast” (*i.e.* non-annealed) state. Thermally annealing these films above 80 °C can considerably increase ellipticity in the F8BT absorption band, reaching over 4000 mdeg at an optimal annealing temperature of approximately 130 °C.^31^

**Fig. 2:**
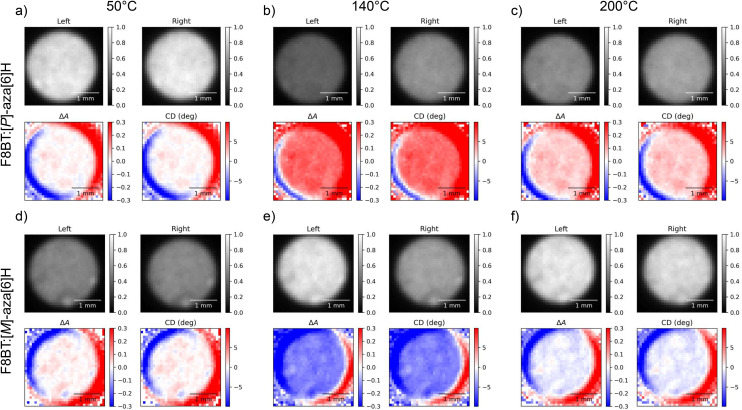
Circular dichroism images of 140 nm thick solid state F8BT:[*P*]-aza[6]H (a, b, c) and F8BT:[*M*]-aza[6]H (d, e, f) films at annealing temperatures of 50 °C (a, d), 140 °C (b, e), and 200 °C (c, f) taken using the two camera circular dichroism imaging system at a wavelength of 470 nm. In each subfigure, “Left” and “Right” indicate the transmitted intensities of L-CPL and R-CPL (respectively), Δ*A* is calculated using eqn (5) and “CD” is the degrees of ellipticity calculated using eqn (2).

Fig. 2 visualises the outputs of the 2-CCD*i* system for F8BT:[*P*]- and [*M*]-aza[6]H films (*λ*_ex_ = 470 nm) as a function of temperature (50 °C to 200 °C at a rate of 10 °C per minute). The Linkam chamber used for the temperature and environmental control of heated samples has a circular aperture of 2.5 mm, which limits the size of the mapped area to this diameter. This may be improved through the use of a custom chamber with a larger transmission aperture or through the use of a Linkam stage with sample translation stage. Videos of example annealing cycles are included in the ESI.†

**Fig. 3:**
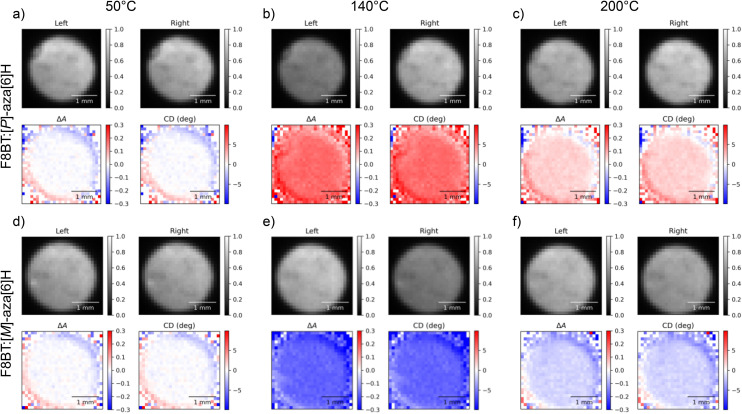
Circular dichroism images of 140 nm thick solid state F8BT:[*P*]-aza[6]H (a, b, c) and F8BT:[*M*]-aza[6]H (d, e, f) films at annealing temperatures of 50 °C (a, d), 140 °C (b, e), and 200 °C (c, f) taken using the one camera circular dichroism imaging system at a wavelength of 470 nm. In each subfigure, “Left” and “Right” indicate the transmitted intensities of L-CPL and R-CPL (respectively), Δ*A* is calculated using eqn (5) and “CD” is the degrees of ellipticity calculated using eqn (2).

Remarkably, the CD response as a function of temperature is consistent with data acquired in our previous study of F8BT:aza[6]H blends using the CD*i* capabilities of the Diamond Light Source (DLS).^31^ Specifically, F8BT:[*M*]- and [*P*]-aza[6]H show negative and positive ellipticity, respectively, of equal magnitude at each annealing temperature. Further, the relationship between the annealing temperature and the magnitude of ellipticity is consistent with our previous studies, increasing monotonically from 50 °C to a maximum ellipticity at 140 °C (indicating the formation of a chiral phase with a strong chiroptical response), above which the chiral phase is destroyed and ellipticity decreases. In an attempt to align the images of the two cameras we used both an optical cage system and post-processing, but minimal misalignment and reflection from the internal surface of the Linkam chamber aperture results in circular artefacts which appear as a red and blue “halo” around the circular edge of each sample image. To mitigate for these artefacts, we performed the same experiment using the 1-CCD*i* system (Fig. 3, full cycle in ESI†). As shown, the use of a single camera in the 1-CCD*i* reduces the size of this halo artefact due to the absence of misalignment between two CMOS sensors.

**Fig. 4:**
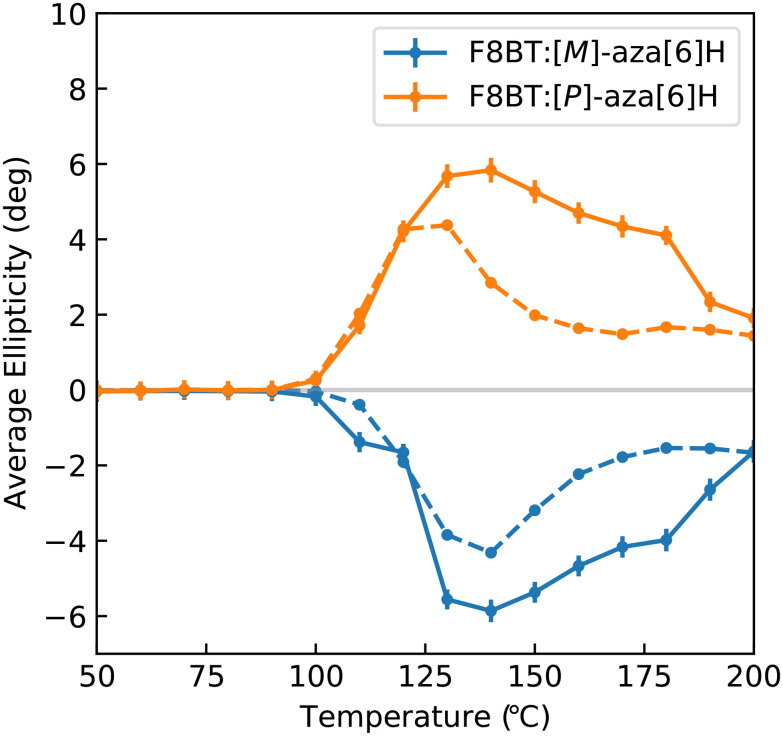
Spatially averaged ellipticity of solid state F8BT:[*M*]-aza[6]H and F8BT:[*P*]-aza[6]H samples measured using the one camera system (solid line) and using Beamline 23 at the Diamond Light Source (dashed line) at a wavelength of 470 nm. Differences between these data sets are discussed in the Experimental section. Data from the Diamond Light Source has been reproduced from ref. 36 under a Creative Commons Attribution 4.0 International License.

Fig. 4 illustrates the average ellipticity of the F8BT:[*M*]- and [*P*]-aza[6]H films as a function of temperature for the 1-CCD*i* system, which can be calculated by averaging individual pixels within the CMOS array. Promisingly, these results are highly consistent with those reported in our previous work,^31^ with ellipticity increasing significantly above 90 °C to a maximum at 130–140 °C before gradually decreasing at higher annealing temperatures. Differences between these data sets are discussed in the Experimental section. As these measurements are quick to perform and the instrumentation is inexpensive and compact, it would be trivial to perform measurements during sample preparation. This would allow for fabrication protocols (*e.g.* solvent choice, spin-coating speed, *etc*.) to be refined and optimised.

To estimate the weakest detectable chiroptical response, we calculated the standard deviation of the pixel values used to generate Fig. 4 for *T* < 90 °C (that is, no detectable chiroptical response). These results indicate that the lowest CD value that we can reliably measure is ∼250 mdeg. At this current CD sensitivity, this system is suitable for the study of high ellipticity samples (*e.g.* supramolecular assemblies) and liquid crystalline materials (*e.g.* cellulose nanocrystals).^7^ To improve the sensitivity to weak chiroptical responses, the cameras could be replaced with a thermoelectrically cooled CMOS array and a compact camera-mounted enclosure with an integrated sample holder to eliminate noise contributions from external light sources.

**Fig. 5:**
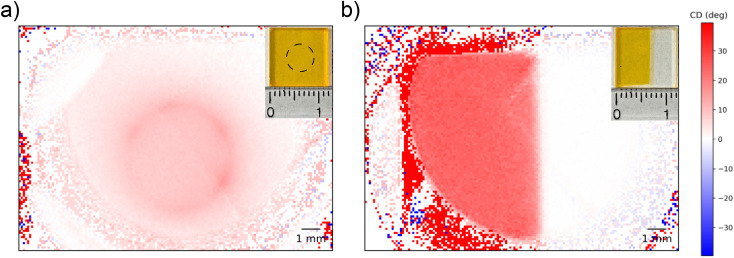
Spatially patterned solid state films of thermally annealed F8BT:[*P*]-aza[6]H with ring (a) and step (b) profiles (see Experimental section 6.2). Images were captured using the 1-CCD*i* system in ambient conditions at a wavelength of 470 nm and using a conventional visible light camera (inset, scale in cm). Note that the circular pattern (a) is not noticeable using the visible light camera, and as such, the intended patterning shape is indicated by a dotted line overlay.

To test the spatial resolution of the 1-CCD*i* system we consider two F8BT:[*P*]-aza[6]H thin films which were intentionally patterned using thermal and solvent annealing (see Experimental section) to produce domains of strong/weak chiroptical response (Fig. 5). As shown, features could be easily resolved using the low-cost cameras and, due to being measured outside the Linkam chamber in air, show no halo artefacts. We performed measurements on the same samples using highly collimated light from the DLS and found good agreement (ESI, Fig. S4†), with a spatial resolution of 100 μm. At this resolution, this imaging system can be used to determine the spatial uniformity of chiroptical films typically used in the fabrication of CPL sensitive organic photodetetectors and CPL emitting OLEDs (approximately 10 mm × 10 mm) or could be used to study spatially mapped chiroptical samples with a minimum feature size of 100 μm (*e.g.* for banknote counterfeit screening). This resolution may be further improved through the use of microscope optics, and we hope that further resolution improvements will enable the study of chiral plasmonic nanostructures and the morphology of chiral supramolecular structures.^37–39^

Linear birefringence (LB) and linear dichroism (LD) can lead to orientation-dependent artefacts in the measured CD of chiroptical materials due to the misinterpretation of linearly polarised light as CPL by the imaging system.^18,26^ LD and LB are revealed as changes in the measured CD when the film is rotated about the optical axis of the CD spectrometer or CD imaging system.^18^ Coupling between LB and LD (often termed the LDLB effect), on the other hand, leads to true selective absorption of CPL (*i.e.*, CD) which also has directional dependence.^26^ The LDLB effect may be identified by a change (or in some cases inversion) of the CD when measurements are made with the film facing toward and away from the detector.^26^

**Fig. 6:**
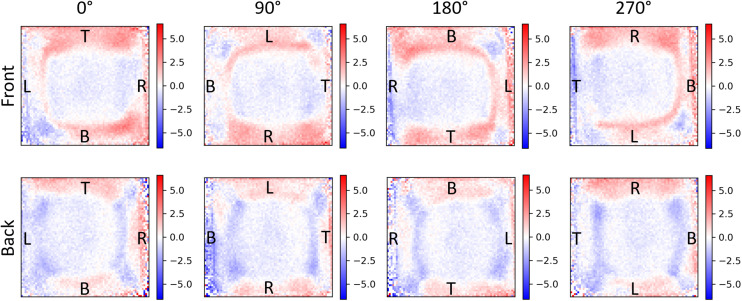
Ellipticity images (in units of degrees) of cellulose nanocrystals (CNCs) captured using the 1-CCD*i* system in ambient conditions at a wavelength of 405 nm. Each subfigure shows the same CNC film viewed from the front or back (*i.e.* through the quartz substrate) of the film at various angles of rotation around the optical axis of the camera system. To enable straightforward comparison between rotated images, the left, right, top, and bottom edges of the CNC film at 0° are marked by L, R, T, and B, respectively.

Cellulose nanocrystals (CNCs) are known to form chiral cholesteric nematic liquid crystal phases on drying, owing to the intrinsic left-handed twist present in naturally occurring cellulose.^18^ Prior work by Hussain *et al.* using the CD imaging capabilities at the DLS has demonstrated LD and LB in CNC films leading to orientation-dependent CD artefacts.^18^ To further benchmark our own CD imaging system, we evaluated whether our 1-CCD*i* can also identify LD, LB, and the LDLB effect in chiroptical systems (Fig. 6 and S5†). These CNC films show no appreciable change in measured CD when the sample is rotated or flipped when measured using a conventional CD spectrometer (Fig. S6†). Imaging the sample using the 1-CCD*i* system, however, not only shows the underlying non-uniformity of the CD of the CNC film, but we can also show that the spatial variation of CD is sensitive to rotation of the film (indicating the presence of spatially varying LD and LB in the film) and flipping of the film (demonstrating the LDLB effect in this film).

## 5 Conclusions

We have designed and built a highly versatile low-cost, compact and modular chiroptical imaging system for the analysis of chiral functional materials that is capable of detecting CD as weak as 250 mdeg and has a spatial resolution of 100 μm. We have also developed open-source Python control software for this system which is available at our GitHub repository https://github.com/rmdocherty/CPL-Imager.

Presently, the chiroptical response is characterised at a single excitation wavelength, but a tunable monochromatic light source would allow for measurements over a full spectral range. Whilst we have presented this apparatus as a means to characterise the CP absorbance (*i.e.* CD measurements), with appropriate filters, spatially resolved CP luminescence could also be acquired (as demonstrated by Baguenard *et al.*^40^), making this instrument an “all-in-one” portable chiroptical probe.

This instrument continues to be under active development, with new features being added regularly. We anticipate being able to improve the sensitivity of this system through the use of a compact, camera-mounted chamber for sample mounting to block stray light sources. We are also in the process of an in-depth analysis of potential sources of noise in these measurements, such as light source stability and dark shot noise in our camera. In both cases, noise may be suppressed by using active temperature control, such as a water- or fan-cooled light source and a thermoelectrically cooled CMOS array camera. With these modifications and the use of microscope optics, we aim to improve the spatial resolution and sensitivity of this system to below 100 μm and 100 mdeg, respectively. Through the use of a custom chamber with a larger transmission aperture or in conjunction with a sample translation stage, it is possible to increase the area of the sample under test which can be imaged.

The basic camera systems developed in this work lower the barriers to entry to studying chiroptical semiconductors, such as equipment cost, facility access, and instrumentation expertise. We hope that lowering these barriers will encourage other research groups to contribute to the exciting and rapidly growing field of chiral organic materials.

## 6 Experimental section

### 6.1 F8BT:aza[6]H solution preparation and thin film deposition

F8BT (*M*_w_ = 31 kDa) and aza[6]H were dissolved in toluene to a concentration of 20 mg ml^−1^ and blended to form a 10% (wt%) aza[6]H solution. The achiral polymers were provided by Cambridge Display Technology (CDT). Fused-silica substrates were rinsed in an ultrasonic bath in a three-step process using acetone, isopropyl alcohol and Hellmanex™ III for 15 min each. They were then transferred to an oxygen plasma asher for 3 min at 80 W before spin-coating. Thin films were spin-coated at 2000 rpm for 60 s to produce films with an average thickness of 140 nm. Non-patterned thin films were then annealed in a nitrogen glovebox, with <0.1 ppm H_2_O and O_2_.

### 6.2 Spatially patterned F8BT:aza[6]H thin films

First, homogeneous as-cast F8BT:[*P*]-aza[6]H thin films were deposited as detailed in *Solution preparation and thin film deposition* above, except by instead using a 35 mg ml^−1^ solution concentration. Two different routes were applied to achieve spatial pattern domains with strong chiroptical activity. Route (1): as-cast thin films were turned over and carefully placed face down onto a ring-shaped metal piece (of ∼4 mm diameter) that was heated at 160 °C, for 10 min. Such a process yields samples like the one presented in Fig. 5a, with a thickness of 285 ± 2 nm. Route (2): 1.5 mm thick viscoelastic PDMS layers were site-selectively masked onto the as-cast thin films, creating well-adhered solvent barriers for later processing. The thin films with PDMS masks were loaded back onto a spin-coater, followed by dispensing ∼100 μL toluene. Toluene was thus allowed to cover the samples for ∼15 s prior to its removal as the spin-coating process initiated. This method preserves thin film content underneath the masked area(s) and removes the material elsewhere, exposing the bare fused-silica substrate. For chiral induction in the polymer phase, PDMS masks were carefully lifted off prior to a thermal annealing treatment performed at 140 °C for 10 min. Samples were then immediately quenched onto a cold (∼20 °C) metal surface. Thin films patterned this way include the ones presented in Fig. 5b, with a thickness of 257 ± 5 nm.

### 6.3 Cellulose nanocrystal films

Fused-silica substrates were first cleaned and plasma ashed using the same method described above for the fabrication of F8BT:aza[6]H thin films. A 6 wt% cellulose nanocrystal water slurry provided by Cellulose Lab was then diluted to 3% wt and drop-cast onto the cleaned fused-silica substrates. Drop-cast films were dried for 48 hours at room temperature in cleanroom conditions before CD imaging and CD spectroscopy were performed on these samples. The thickness of the drop-cast films varies significantly across the surface of the film, with an average thickness of 622 ± 98 nm.

### 6.4 Dual and single camera circular dichroism imaging systems

The single and dual camera systems both made use of a 470 nm, 809 mW mounted LED (Thorlabs, M470L5) to illuminate the sample under test. This LED was filtered with a 470 ± 10 nm bandpass filter (Thorlabs, FBH470-10) and collimated using an aspheric condenser lens with diffuser (Thorlabs, ACL2520U-DG6-A) mounted in a lens tube system (Thorlabs, SM1V05, SM1L03). Left- and right-handed circularly polarised light transmitted through the sample under test is converted to two orthogonal linear polarisations (vertical and horizontal) using a mounted quarter-wave Fresnel rhomb retarder (Thorlabs, FR600QM). In the dual-camera system, the vertical and horizontal linear polarisations are directed to two separate cameras by a polarising beamsplitter cube (Thorlabs, CCM1-PBS251/M). In the single-camera setup, transmission of the vertical and horizontal polarisations to a single camera are rapidly switched between using a wire grid polariser (Thorlabs, WP25M-VIS) mounted in a piezoelectric rotation mount (Thorlabs, ELL14K). For both dual- and single-camera imaging systems, USB controlled monochrome Zelux® 1.6 MP CMOS cameras are used (Thorlabs, CS165MU/M), with images focused onto the camera CMOS arrays using a 6 mm focal length *f* = 1.4 Navitar lens (Thorlabs, MVL6WA). A price list of all components used in the single- and dual-camera circular dichroism imaging systems has been provided in the ESI.†

### 6.5 Thermal annealing

*In situ* thermal annealing of solid-state samples during CD imaging was controlled by a temperature controlled stage (Linkam, LTS40 and T96). The temperature range, ramp rate and dwell time was pre-programmed using a Linkam Linkpad touchscreen controller. In all cases, the chamber heating rate was 10 °C min^−1^ and the samples were held at a given temperature for 1 min before CD images were captured. In the case of thermal annealing measurements performed using the DLS (Fig. 4), the heating rate and holding time for a given temperature were identical, however, in this case, full CD spectra in the range of 200–650 nm were obtained at each temperature. Measurements of complete spectra increase the measurement time to approximately 7 minutes (the monochromator scanning rate and detector integration time), which increases the effective annealing time at each temperature when compared to single wavelength measurement obtained using the 1-CCD*i*.

### 6.6 Control software

The control software was written in the Python programming language, utilising the Thorlabs software interface for compact scientific cameras. The result was a multi-threaded program that allows for real-time image acquisition from the connected camera or cameras. For the 1-CCDI design, the software also controls the piezoelectric motor, ensuring successive images are of alternating handedness. A Graphical User Interface (GUI) then enables the visualisation and measurement of *A*_L_ and *A*_R_ either side-by-side or combined as Δ*A* or ellipticity, either in photos or using the cursor for reading values at a point. A switchable overlay shows the sizes of objects in the photos; a screenshot demonstrating these GUI functions is available in ESI (Fig. S7†). Calibration options allow for selecting regions-of-interest of the image, first-order setup corrections, and defining the pixel-per-millimetre ratio. The programme is open-source, and a link to a GitHub repository containing the source code is provided here: https://github.com/rmdocherty/CPL-Imager.

## Conflicts of interest

The authors declare the following competing financial interest(s): M. Fuchter is an inventor on a patent concerning chiral blend materials (WO2014016611).

## Supplementary Material

NR-016-D4NR01651C-s001

NR-016-D4NR01651C-s002

NR-016-D4NR01651C-s003

NR-016-D4NR01651C-s004

NR-016-D4NR01651C-s005
