# Supplementary material for: Development of low-cost, compact chiroptical imaging systems
Source: Nanoscale. 2024 Jun 4;16(24):11623–32. doi: 10.1039/d4nr01651c (PMC11189636; doi:10.1039/d4nr01651c)
Supplement: NR-016-D4NR01651C-s005 [file NR-016-D4NR01651C-s005.pdf]

Electronic Supplementary Material (ESI) for Nanoscale.
This journal is © The Royal Society of Chemistry 2024

Supporting Information

Development of low-cost, compact chiroptical imaging systems

Matthew D. Ward,[‡a,b] Ronan Docherty,[‡a] Louis Minion,[b,d,e] Xingyuan Shi,[b,c] Kai Anson,[a] Giuliano Siligardi,[e] Jenny Nelson,[a,b] Jessica Wade,[*b,d] and Matthew J. Fuchter[*b,c]

Dedication: *Dedicated to the memory of Professor Alasdair James Campbell*

[a]*Department of Physics, Imperial College London, South Kensington Campus, Prince Consort Road, London SW7 2AZ, UK.*

[b]*Centre for Processable Electronics, Imperial College London, South Kensington Campus, London SW7 2AZ, UK.*

[c]*Department of Chemistry and Molecular Sciences Research Hub, Imperial College London, White City Campus, 82 Wood Lane, London W12 0BZ, UK. E-mail: m.fuchter@imperial.ac.uk*

[d]*Department of Materials, Exhibition Road, Imperial College London, SW7 2AZ. E-mail: jessica.wade@imperial.ac.uk*

[e]*B23 Beamline, Diamond Light Source Ltd, Harwell Science and Innovation Campus, Didcot, UK*

‡ *These authors contributed equally to this work*

Derivation of Ellipticity

The electric field of elliptically polarised light (purple ellipse, Figure S1) may be considered an unequal sum of the electric field of a left- (red circle, Figure S1) and right-handed circularly polarised electric field (blue circle, Figure S1).

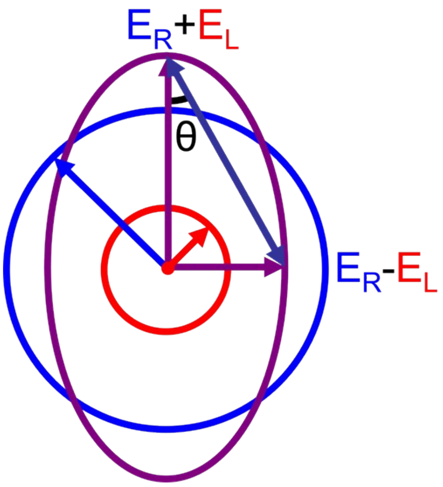

Figure S1 *The electric field of elliptically polarised light (purple) expressed as the sum of a left- (red) and right-handed (blue) circularly polarised electric field. The ellipticity of the resultant electric field is shown here as θ.*

The ellipticity of a given polarisation, θ, is defined as the angle between the points on the ellipse which intersect the major and minor axes. This is given by

$$Tan\,\theta = \frac{E_R - E_L}{E_R + E_L} \tag{S1}$$

where E_L and E_R are the magnitudes of the electric fields corresponding to the left- and right-handed circularly polarised components of the elliptical polarisation. This may be expressed in terms of the intensity of the left- and right-handed circularly polarised light as follows:

$$Tan\,\theta = \frac{I_R^{\frac{1}{2}} - I_L^{\frac{1}{2}}}{I_R^{\frac{1}{2}} + I_L^{\frac{1}{2}}} \tag{S2}$$

where we have used the relation

$$E \propto I^{\frac{1}{2}}. \quad \text{(S3)}$$

For a sample illuminated by unpolarised or linearly polarised light, the incident intensity of left- and right-handed circularly polarised light are equal (I_0). For an optically thick absorbing sample (i.e., a sample without optical interference effects), the intensity of left- or right-handed circularly polarised light transmitted through the sample is given by

$$I = I_0 e^{-A\ln(10)} \quad \text{(S4)}$$

where A is the absorbance of sample. Applying this result to equation S2 we find:

$$\begin{aligned}
Tan\,\theta &= \frac{I_R^{\frac{1}{2}} - I_L^{\frac{1}{2}}}{I_R^{\frac{1}{2}} + I_L^{\frac{1}{2}}} \\
&= \frac{I_0 e^{-A_R\frac{\ln(10)}{2}} - I_0 e^{-A_L\frac{\ln(10)}{2}}}{I_0 e^{-A_R\frac{\ln(10)}{2}} + I_0 e^{-A_L\frac{\ln(10)}{2}}} \\
&= \frac{e^{-A_R\frac{\ln(10)}{2}} - e^{-A_L\frac{\ln(10)}{2}}}{e^{-A_R\frac{\ln(10)}{2}} + e^{-A_L\frac{\ln(10)}{2}}} \\
&= \frac{e^{(A_L - A_R)\frac{\ln(10)}{2}} - 1}{e^{(A_L - A_R)\frac{\ln(10)}{2}} + 1} \\
&= \frac{e^{\Delta A\frac{\ln(10)}{2}} - 1}{e^{\Delta A\frac{\ln(10)}{2}} + 1} \\
&= \tanh\left(\frac{\Delta A\ln(10)}{4}\right)
\end{aligned} \quad \text{(S5)}$$

$$\theta = \tan^{-1}\left\{\tanh\left(\frac{\Delta A\ln(10)}{4}\right)\right\}[rad] \quad \text{(S6)}$$

where A_R and A_L are the absorbances of the sample under right- and left-handed circularly polarised light and ΔA is the difference between A_R and A_L (i.e., $A_L - A_R$). Finally, this may be converted from radians to the common ellipticity units of degrees, yielding:

$$\theta = \tan^{-1}\left\{\tanh\left(\frac{\Delta A \ln(10)}{4}\right)\right\}\frac{180}{\pi}\,[deg] \qquad \text{(S7)}$$

For small values of ΔA, this simplifies to:

$$\theta \approx \frac{\Delta A \ln(10)}{4}\frac{180}{\pi} = 32.982\,\Delta A\,[deg] \qquad \text{(S8)}$$

Figure S2 demonstrates the agreement between the precise and approximate equations for ellipticity as a function of ΔA. As shown, the percentage deviation between the two equations remains less than 1% for $\Delta A < 0.213$, but this deviation increases significantly above this value.

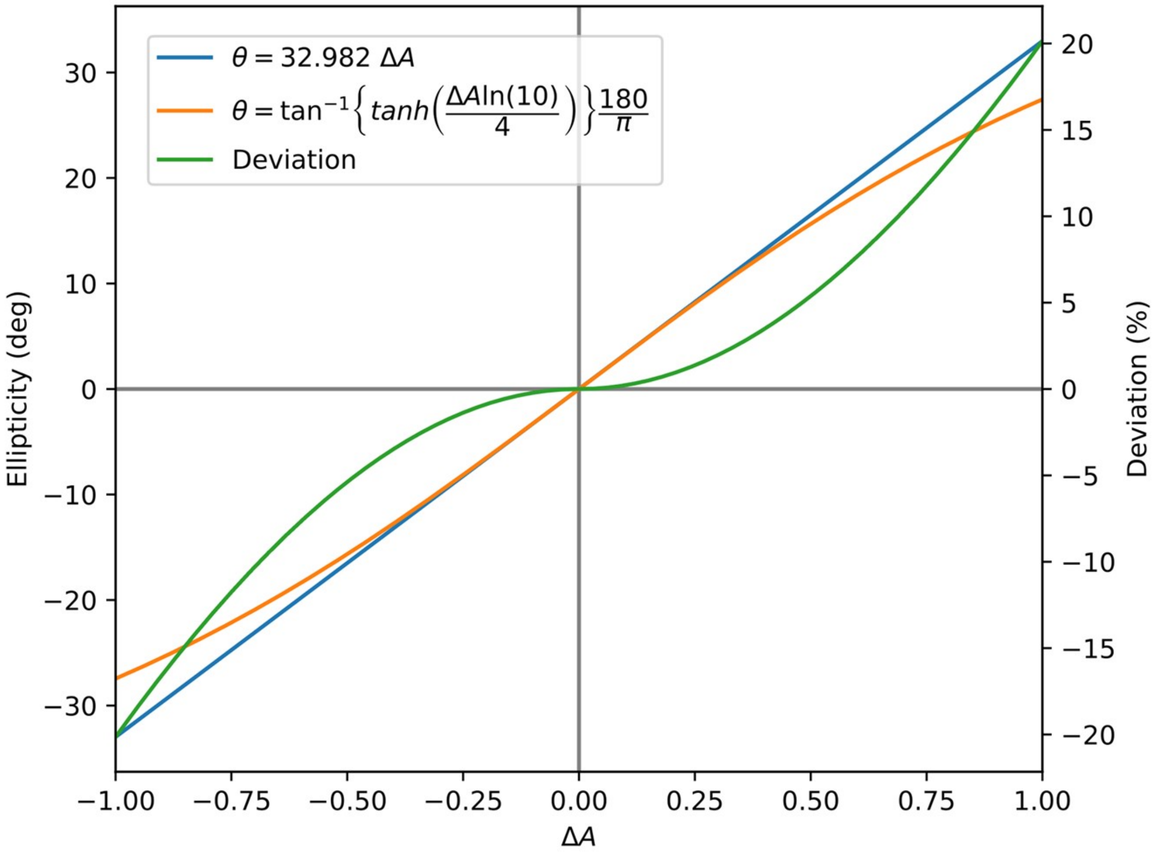

Figure S2 *The precise (orange) and approximate (blue) equations for ellipticity expressed as a functions of* ΔA. *The percentage deviation (green) of the approximate expression from the precise expression becomes considerable (>1%) for* $\Delta A > 0.213$.

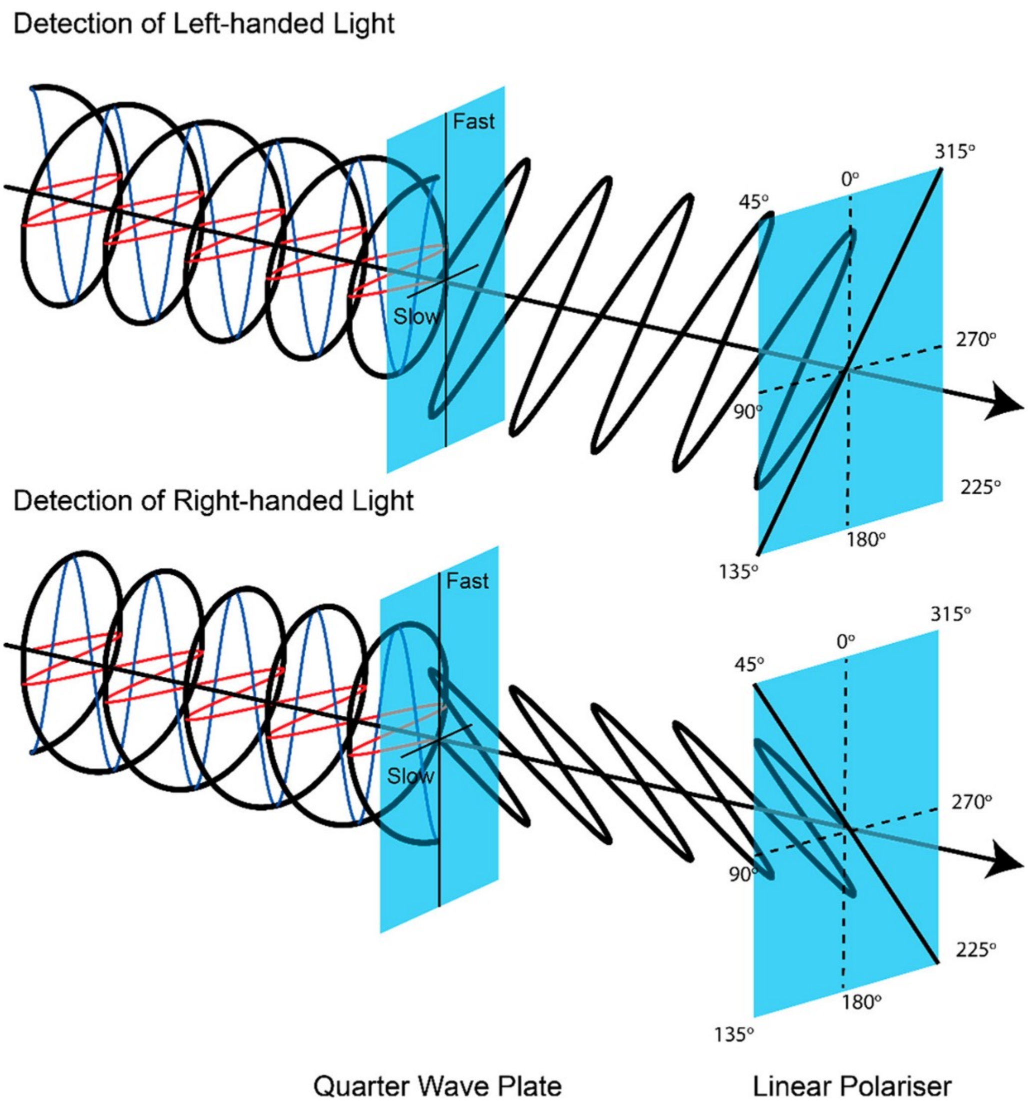

Figure S3 *Conventions for left- and right-handed circualrly polarised light used in this work.*

Measurement of Ellipticity and Absorption Dissymmetry

As discussed in the manuscript, ΔA is calculated as follows:

$$\Delta A = A_L - A_R$$

$$= \log_{10}\left(\frac{I_0}{I_L}\right) - \log_{10}\left(\frac{I_0}{I_R}\right)$$

$$= \log_{10}(I_0) - \log_{10}(I_L) - \log_{10}(I_0) + \log_{10}(I_R)$$

$$= \log_{10}(I_R) - \log_{10}(I_L)$$

$$= \log_{10}\left(\frac{I_R}{I_L}\right)$$

I_L and I_R are the intensities of left- and right-handed circularly polarised light transmitted through the sample under test and I_0 is the intensity of either left- or right-handed circularly polarised light incident on the sample. The above equation demonstrates that the initial intensity of light does not need be

measured for ΔA to be calculated – only the transmitted intensities (as measured by the cameras) are required.

Similarly, for g_{abs}:

$$g_{abs} = 2\frac{A_L - A_R}{A_L + A_R}$$

$$= 2\frac{\Delta A}{A_L + A_R}$$

$$= 2\frac{\log_{10}\left(\frac{I_R}{I_L}\right)}{\left[\log_{10}\left(\frac{I_0}{I_L}\right) + \log_{10}\left(\frac{I_0}{I_R}\right)\right]}$$

$$= 2\frac{\log_{10}(I_R) - \log_{10}(I_L)}{\left[\log_{10}(I_0) - \log_{10}(I_L) + \log_{10}(I_0) - \log_{10}(I_R)\right]}$$

$$= 2\frac{\log_{10}(I_R) - \log_{10}(I_L)}{\left[2\log_{10}(I_0) - \log_{10}(I_L) - \log_{10}(I_R)\right]}$$

Measurements of g_{abs} require measurements of I_0, I_R and I_L – the measurement of incident intensity cannot be avoided, unlike the case of ΔA. While the argument could be made that a calibration measurement could be made beforehand using a blank substrate to enable the measurement of I_0, this does not account for:

- Any differences between the position of the sample and the substrate, especially given the 100 µm spatial resolution of the system.
- Any changes in light intensity following the initial calibration. Often the intensity of the illumination source must be modified during annealing to account for changes in absorption due to phase changes within the sample.

For this reason, measurements of g_{abs} obtained using a calibration sample will suffer from considerably more uncertainty than that of a measurement of ellipticity.

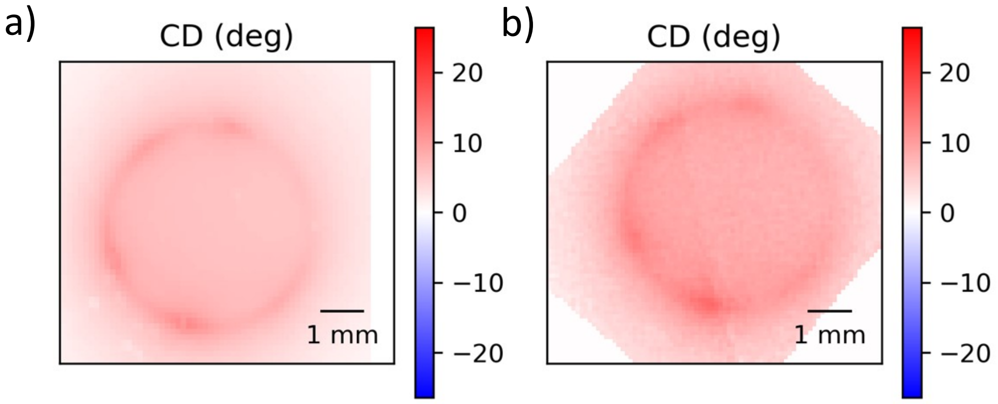

Figure S4 *A direct cross-comaprison between CD images of spatially patterned solid state F8BT:[P]-aza[6]H captured using beamline 23 at the diamond light source (a) and the one camera CD imaging system (b).*

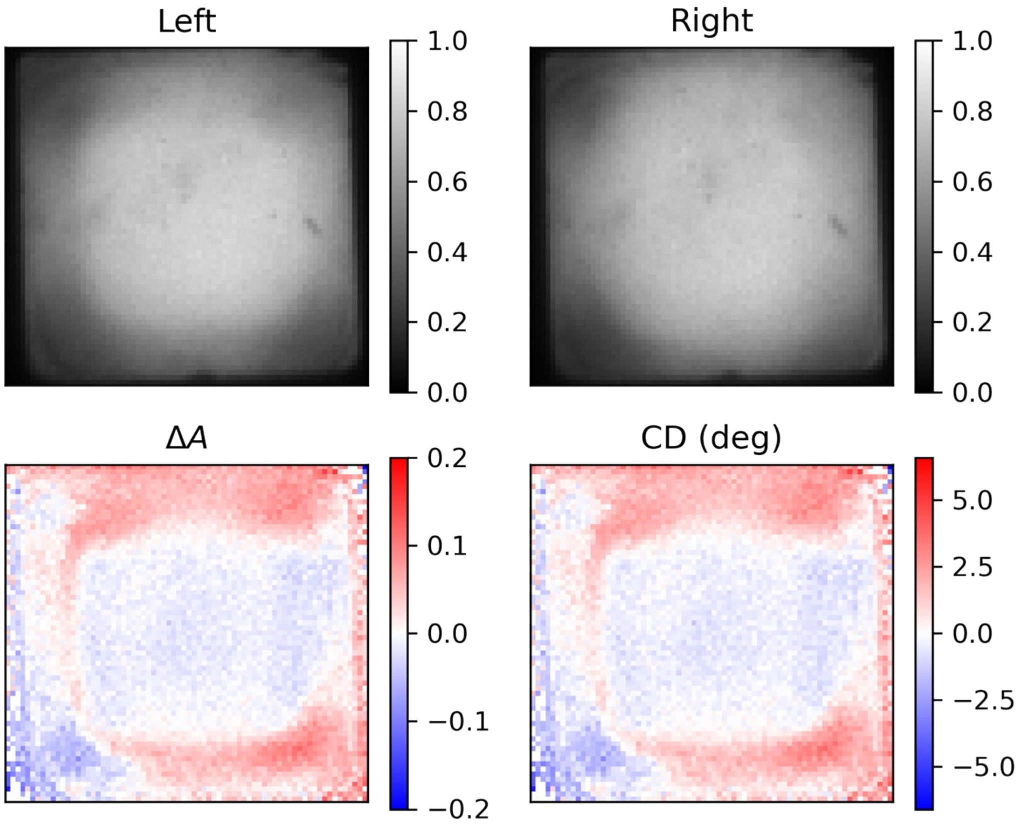

Figure S5 *An image of the un-rotated (0°) and un-flipped (front) cellulose nanocrytsal film taken using the 1-CCDI system at a wavelength of 405 nm.*

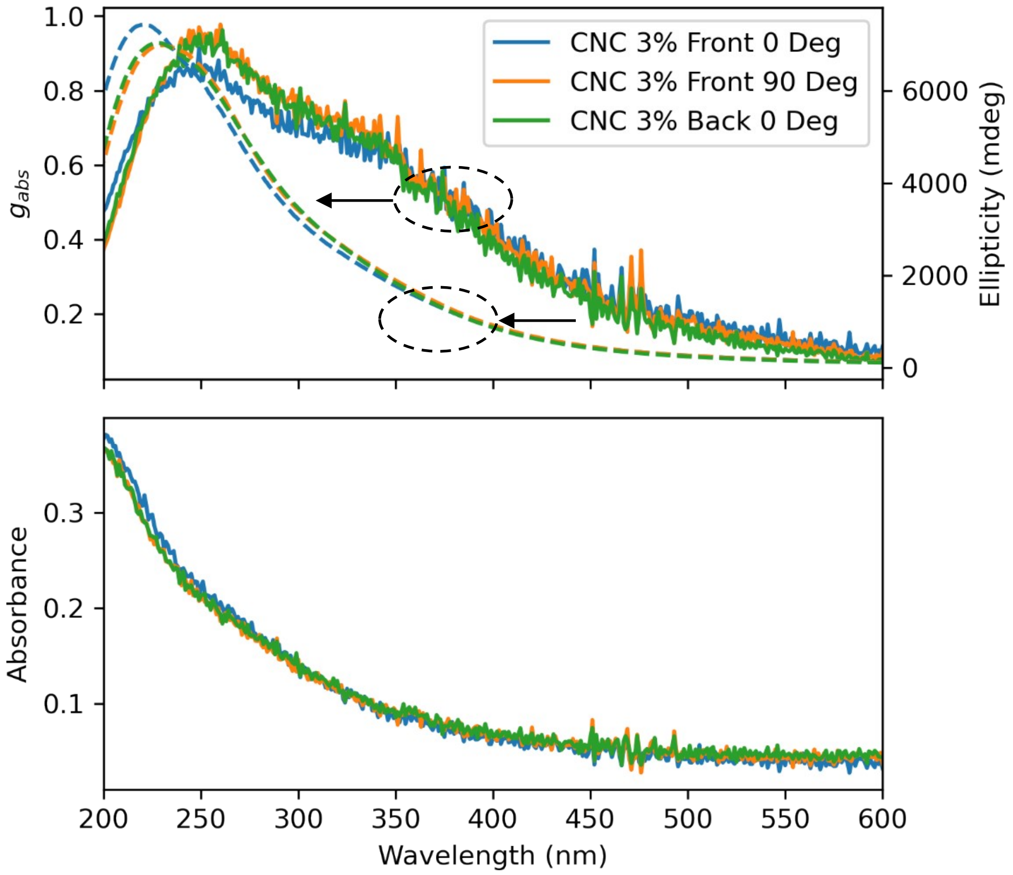

Figure S6 *CD,* g_{abs}*, and absorption spectra of the cellulose nanocrystal film under rotation around the optical axis and sample flipping. At the wavelength of interest (405 nm), almost no change in any of the measured parameters is observed.*

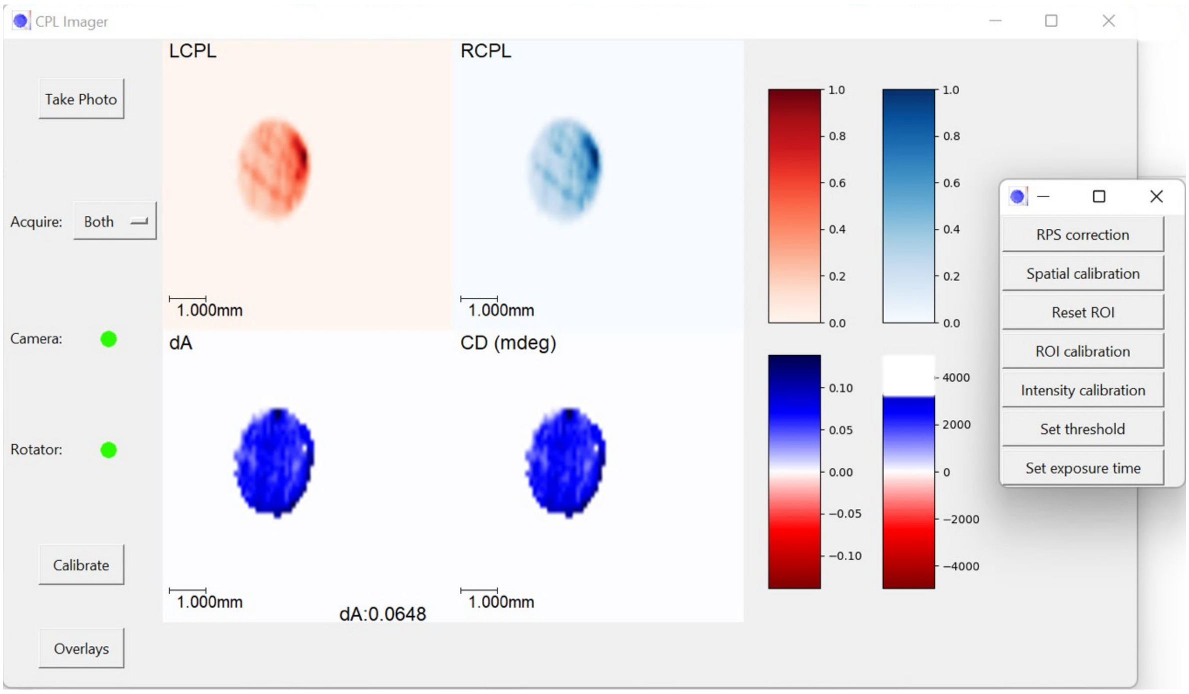

Figure S7 *A screenshot of the custom open-source python graphical user interface used to control the one- and two-camera circular dichroism imagers.*

Table S1 *List of parts used to assemble the two- and one-camera CD imaging systems. Prices are accurate at the time of writing, September 2022.*

**2 Camera System**		
**Part Number**	**Description**	**Price**
CS165MU/M	Zelux® 1.6 MP Monochrome CMOS Camera, M6 Taps	£336.04
CS165MU/M	Zelux® 1.6 MP Monochrome CMOS Camera, M6 Taps	£336.04
CCM1-PBS251/M	30 mm Cage Cube-Mounted Polarizing Beamsplitter Cube, 420-680 nm, M4 Tap	£253.72
MVL6WA	6 mm EFL, f/1.4, for 1/2" C-Mount Format Cameras, with Lock	£124.79
MVL6WA	6 mm EFL, f/1.4, for 1/2" C-Mount Format Cameras, with Lock	£124.79
FR600QM	Mounted Quarter-Wave Fresnel Rhomb Retarder	£313.62
**Total**		**£1,489.00**

**1 Camera System**		
**Part Number**	**Description**	**Price**
CS165MU/M	Zelux® 1.6 MP Monochrome CMOS Camera, M6 Taps	£336.04
MVL6WA	6 mm EFL, f/1.4, for 1/2" C-Mount Format Cameras, with Lock	£124.79

ELL14K	Rotation Mount Bundle: ELL14 Mount, Interface Board, Power Supply, Cables	£420.45
WP25M-VIS	Ø25.0 mm Mounted Wire Grid Polarizer, 420-700 nm	£223.78
FR600QM	Mounted Quarter-Wave Fresnel Rhomb Retarder	£313.62
**Total**		**£1,418.68**

**Light Source (with Filter and Collimation Kit)**		
**Part Number**	**Description**	**Price**
M470L5	470 nm, 809 mW (Min) Mounted LED, 1000 mA	£170.94
FBH470-10	Premium Bandpass Filter, Ø25 mm, CWL = 470 nm, FWHM = 10 nm	£115.31
ACL2520U-DG6-A	Aspheric Condenser Lens w/ Diffuser, Ø25 mm, f=20.1 mm, NA=0.60, 600 Grit, ARC: 350 nm - 700 nm	£23.71
SM1V05	Ø1" Adjustable Lens Tube, 0.31" Travel Range	£23.37
SM1L03	SM1 Lens Tube, 0.30" Thread Depth, One Retaining Ring Included	£9.39
**Total**		**£342.72**

**2 Camera System and Light Source**	**£1,831.72**
**1 Camera System and Light Source**	**£1,761.40**
